# Diversity and Impact of Rare Variants in Genes Encoding the Platelet G Protein-Coupled Receptors

**DOI:** 10.1160/TH14-08-0679

**Published:** 2015-01-08

**Authors:** Matthew L. Jones, Jane E. Norman, Neil V. Morgan, Stuart J. Mundell, Marie Lordkipanidzé, Gillian C. Lowe, Martina E. Daly, Michael A. Simpson, Sian Drake, Steve P. Watson, Andrew D. Mumford, on behalf of the UK GAPP study group

**Affiliations:** 1 School of Cellular and Molecular Medicine, University of Bristol, Level 7 Bristol Royal Infirmary, Bristol, UK; 2 School of Physiology and Pharmacology, University of Bristol, Bristol, UK; 3 Centre for Cardiovascular Sciences, College of Medical and Dental Sciences, University of Birmingham, Birmingham, UK; 4 Centre de recherche de l’Institut de cardiologie de Montréal, and Faculté de pharmacie, Université de Montréal, Quebec, Canada; 5 Department of Cardiovascular Science, University of Sheffield Medical School, Sheffield, UK; 6 Division of Genetics and Molecular Medicine, King’s College, London, UK

**Keywords:** Receptors, G-protein-coupled, genetic variation, blood platelets, blood platelet disorders

## Abstract

Platelet responses to activating agonists are influenced by common population variants within or near G protein-coupled receptor (GPCR) genes that affect receptor activity. However, the impact of rare GPCR gene variants is unknown. We describe the rare single nucleotide variants (SNVs) in the coding and splice regions of 18 GPCR genes in 7,595 exomes from the 1,000-genomes and Exome Sequencing Project databases and in 31 cases with inherited platelet function disorders (IPFDs). In the population databases, the GPCR gene target regions contained 740 SNVs (318 synonymous, 410 missense, 7 stop gain and 6 splice region) of which 70% had global minor allele frequency (MAF) < 0.05%. Functional annotation using six computational algorithms, experimental evidence and structural data identified 156/740 (21%) SNVs as potentially damaging to GPCR function, most commonly in regions encoding the transmembrane and C-terminal intracellular receptor domains. In 31 index cases with IPFDs (Gi-pathway defect n=15; secretion defect n=11; thromboxane pathway defect n=3 and complex defect n=2) there were 256 SNVs in the target regions of 15 stimulatory platelet GPCRs (34 unique; 12 with MAF<1% and 22 with MAF ≥ 1%). These included rare variants predicting R122H, P258T and V207A substitutions in the P2Y_12_ receptor that were annotated as potentially damaging, but only partially explained the platelet function defects in each case. Our data highlight that potentially damaging variants in platelet GPCR genes have low individual frequencies, but are collectively abundant in the population. Potentially damaging variants are also present in pedigrees with IPFDs and may contribute to complex laboratory phenotypes.

## Introduction

G protein-coupled receptors (GPCRs) are seven transmembrane domain proteins that mediate signal transduction from a wide range of extracellular stimuli. GPCRs are expressed widely in haematopoietic and vascular tissues, including platelets, in which they mediate activation signals from agonists such as thrombin (protease activated receptors [PAR] 1 and 4), thromboxane A_2_ (thromboxane A_2_ receptor [TP]), epinephrine (α_2A_-adrenoreceptor) and ADP (P2Y_12_ and P2Y_1_ receptors). Platelets also express G_s_-coupled GPCRs such as the prostacyclin (IP_1_), adenosine 2A (A_2A_) and prostaglandin D_2_ (DP_1_) receptors, which mediate inhibitory signals from prostacyclin, adenosine and PGD_2_ respectively, to suppress platelet activation.

Platelet GPCR activity varies between individuals within the population, in part because of common genetic sequence variants (minor allele frequency (MAF) ≥ 1.0%) near, or within GPCR genes. Examples include the variant rs1472122 (downstream of the P2Y_12_ gene *P2RY12*), which affects ADP-induced platelet fibrinogen binding and P-selectin exposure (1) and the variant rs4311994 (downstream of the α_2A_-adrenoreceptor gene *ADRA2A*), which affects epinephrine-induced platelet aggregation (2). Similar associations have been demonstrated between common variants in the PAR1 (*F2R*), PAR4 (*F2RL3*) and the TP receptor *(TBXA2R*) genes and function of the corresponding GPCRs (3–5). Some common variants also influence susceptibility to cardiovascular disease and responses to anti-platelet drugs (4–6). Since the common GPCR gene variants lie exclusively in non-coding regions, these effects are most likely caused by changes in receptor expression, and not altered receptor function (4, 7).

Although the evidence linking common variants near platelet GPCR genes and GPCR activity is compelling, the individual effect size of common variants is small (2). For other genes, rare (MAF<1%) single nucleotide variants (SNVs), with large individual effect size, provide a greater source of inter-individual genetic variation than common variants (8–10). However, for platelet GPCR genes, descriptions of rare variants affecting platelet function are restricted to SNVs in *P2RY12* and *TBXA2R* in isolated pedigrees with inherited platelet function disorders (IPFD) (11–17). It is likely that the impact of rare GPCR gene variants in the population is much greater than implied from these limited descriptions, but this has not been confirmed by systematic analysis. In order to assess the population diversity and impact of rare SNVs in platelet GPCR genes, we have surveyed and annotated coding and splice region SNVs in public databases of 7595 individuals and in 31 cases with IPFD of unknown genetic basis.

## Materials and methods

### G protein-coupled receptors in human platelets

Class A GPCRs that were listed in the International Union of Basic and Clinical Pharmacology (IUPHAR) GPCR Database (Suppl. Table 1, available online at www.thrombosis-online.com) were selected for analysis if present in the Proteomics Identifications Database (PRIDE), the PlateletWeb resource (Suppl. Table 1, available online at www.thrombosis-online.com) and in the human platelet transcriptome with > 1.0 reads per kilobase of exon model per million mapped reads (18).

### GPCR gene variations in population datasets

We identified coding sequence and splice region (from 3 exonic to 8 intronic nucleotides flanking the exon-intron boundaries) SNVs in the GPCR gene shortlist in the April 2012 Integrated Variant Set release of the 1,000 Genomes project and the NHLBI Exome Sequencing Project (ESP) dataset release number ESP6500, accessed through Ensembl Variation 74 (*H. sapiens* Short Variation GRCh37.p13 dataset) using the BioMart tool (Suppl. Table 1, available online at www.thrombosis-online.com). Nucleotide variations were annotated to the consensus coding sequence (CCDS) database transcript of each platelet GPCR.

### GPCR gene variations in inherited platelet function disorders

Genomic DNA was isolated from peripheral venous blood from a representative sub-group of 31 unrelated cases with IPFD recruited at UK Haemophilia Comprehensive Care Centres to the Genotyping and Phenotyping of Platelets (GAPP) study (ISRCTN 77951167, UK REC 06/MRE07/36) according to previously reported eligibility criteria (19). For all cases, platelet function was evaluated using light transmission aggregation and ATP secretion assays using nine agonists at least two weeks after exposure to 19 drugs known to affect platelet function (19, 20). Genomic DNA was enriched for the target GPCR genes either using a custom made bait library for platelet genes (21) or the Agilent SureSelect All Exon 50Mb kit (Agilent Technologies, Wokingham, UK). Sequence data were captured using an Illumina HiSeq 2000 analyser (Illumina Inc San Diego, CA, USA). Sequence reads were mapped to the reference genome GRCh37.p11, Feb 2009 and SNVs were annotated to the consensus CCDS records using the ANNOVAR tool (Suppl. Table 1, available online at www.thrombosis-online.com). Since the IPFD cases all showed reduced platelet responses, we analysed stimulatory platelet GPCRs and excluded the G_s_-coupled inhibitory GPCRs IP_1_, DP_1_ receptor and A_2A_ receptor. All potentially damaging SNVs were confirmed by PCR amplification of individual exons and direct cycle sequencing.

### Functional annotation of GPCR gene variants using computational algorithms

SNVs that were identified in population databases and in cases with IPFD were classified according to sequence ontology terminology used in Ensembl release 74 (Suppl. Table 1, available online at www.thrombosis-online.com). The likely pathogenicity of each SNV was determined using the MAPP, PhD-SNP, PolyPhen-1, PolyPhen-2, SIFT and SNAP prediction tools on the PredictSNP server (Suppl. Table 1, available online at www.thrombosis-online.com). SNVs were classified as potentially damaging if identified as ‘damaging’ by the PredictSNP meta-analysis tool with a consensus likelihood of > 0.5 (22). Splice region variants were analysed using the Human SpliceFinder tool (Suppl. Table 1, available online at www.thrombosis-online.com) and were classified as potentially damaging if the difference between the splice site prediction scores of the wild type and variant sequences exceeded 30% that of the wild-type sequence (23).

### Manual functional annotation of GPCR gene variants

Missense SNVs were also annotated using a manual strategy in which variants were classified as potentially damaging if any of the following criteria were met:

- The substituted amino-acid was within a functional GPCR sequence motif identified in UniProt (Suppl. Tables 1 and 2, available online at www.thrombosis-online.com).
- The substituted amino-acid, expressed in Ballesteros-Weinstein nomenclature, (24) contributed to inter-helical interactions, the ligand binding pocket or to the G-protein binding sites in the consensus Class A GPCR structure (25) or in published crystal structures for the PAR1(26), A_2A_ (27) or P2Y_12_ receptors (28) (Suppl. Table 2, available online at www.thrombosis-online.com).
- There was published experimental evidence of a change in GPCR function from site-directed mutagenesis in a heterologous system, determined from the GPCRDB resource (Suppl. Table 1, available online at www.thrombosis-online.com).

### Analysis of the P2Y_12_ R122H and V207A variants in transfected cells

R122H and V207A HA-tagged human P2Y_12_ constructs were generated by site-directed mutagenesis (Eurofins MWG Operon, Ebersberg, Germany) and were transfected into either HEK293 or 1321N1 cells according to previously described methods (17). Cell surface P2Y_12_ expression in the transfected cells was determined by enzyme linked immunosorbent assay (ELISA) and by immunofluorescence microscopy using murine anti-HA antibody (HA-11) as described previously (17). P2Y_12_ receptor function was measured by incubating the transfected cells with 1 µM forsoklin (Sigma-Aldrich, Gillingham, UK) to increase basal cAMP levels. The cells were then incubated with 50 µM-10 nM ADP before residual cAMP concentrations were determined in cell lysates by ELISA (Sigma-Aldrich cAMP Enzyme Immunoassay Kit, Gillingham, UK).

## Results

### Identification of GPCRs in human platelets

Using the IUPHAR database, we identified 18 Class A GPCRs with robust evidence of expression in human platelets at transcript and protein levels. The coding regions of the 18 GPCR genes had median length of 1121 kb (interquartile range [IQR] 1043–1248) and median GC content of 56.4% (IQR 49.0–64.6; ► Table 1).

### GPCR gene variations in population datasets

**Figure 1:**
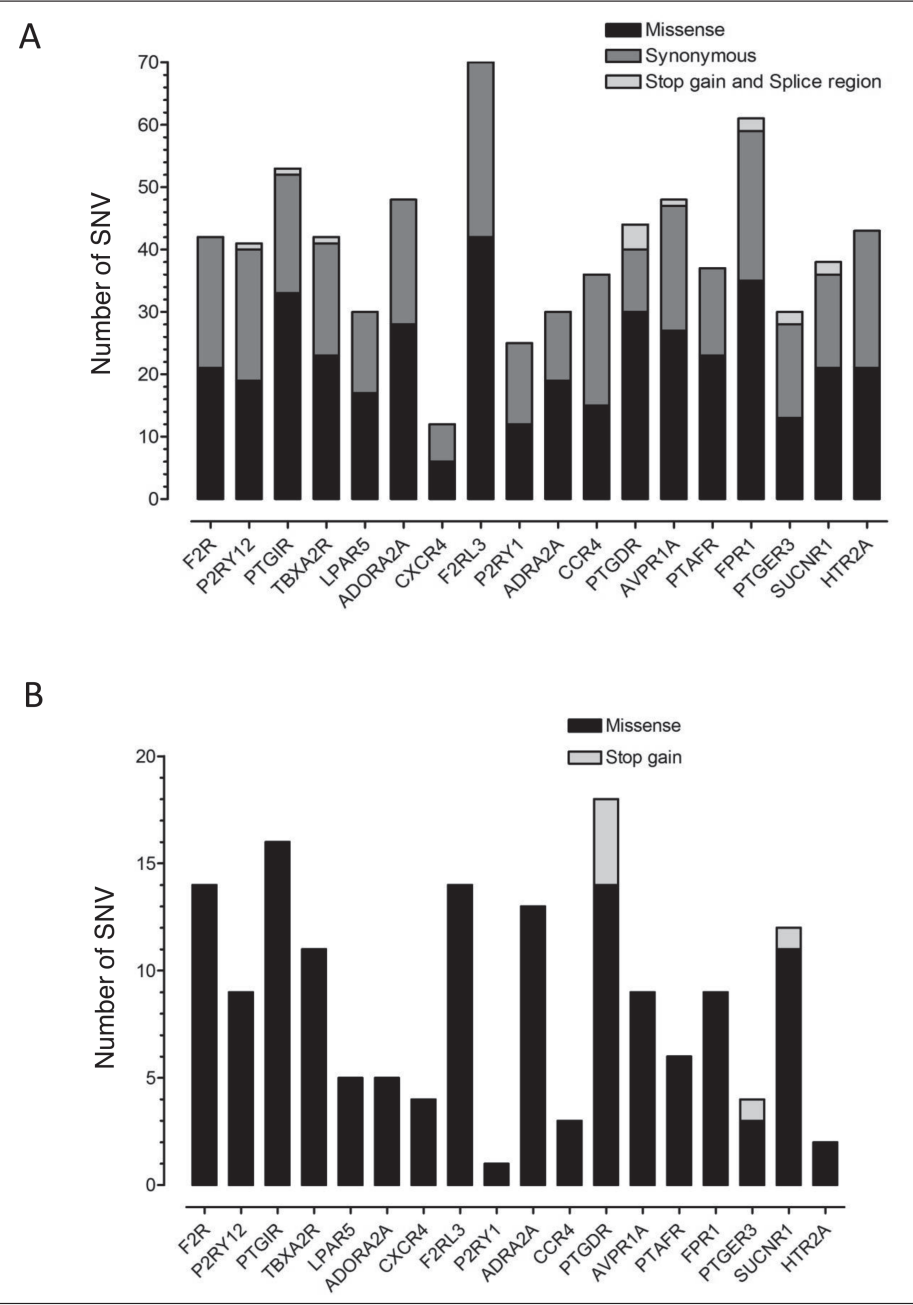
**Distribution of SNVs in the target regions of 18 platelet GPCR genes in the ESP and 1,000 genomes datasets.** A) The total number of unique SNVs in each GPCR gene found in the population datasets, subdivided according to whether missense, synonymous or stop-gain/splice region. B) The total number of unique SNVs in the population datasets that were classified as potentially damaging, subdivided according to whether missense or stop-gain.

In order to assess the allelic diversity of the platelet GPCR genes, we surveyed the 1,000 genomes (1,092 subjects) and ESP (6,503 subjects) datasets for coding and splice region SNVs in the 18 selected GPCR genes. We identified 740 SNVs in the target regions (median 41.5 [IQR 30–48] SNVs per GPCR gene) of which 318 (43%) were synonymous, 410 (55%) were missense and 7 (1%) were stop-gain (► Figure 1A). There were five intronic splice region SNVs and 1 exonic splice region SNV that was also a synonymous coding region SNV. Amongst the 740 SNVs in the target regions, 58 (8%) had global MAF ≥ 1%, 163 (22%) had MAF 0.99–0.05% and 519 (70%) had global MAF<0.05% or were singletons, indicating very low population frequency.

### Predicting the functional impact of GPCR gene variants

We used both computational and manual annotation to assess whether missense SNVs in the GPCR gene target regions were potentially damaging to GPCR function. Computational annotation using the PredictSNP server enabled meta-analysis of predictions from six tools that utilise trained decision (PhD-SNP, Polyphen-2 and SNAP), evolutionary conservation (SIFT), physicochemical (MAPP) and expert rule (Polyphen-1) algorithms to generate a consensus likelihood of pathogenicity for each SNV (22). Using this strategy, 122 (30%) of the 410 missense SNVs in the GPCR gene target regions were classified as potentially damaging (Suppl. Table 3, available online at www.thrombosis-online.com). None of the six splice region SNVs were predicted by computation to disrupt transcript splicing.

Our manual annotation strategy classified missense SNVs as potentially damaging if the predicted amino-acid substitution affected a functional GPCR sequence motif or a critical residue in the consensus or specific GPCR crystal structures or if previous experimental mutagenesis of the residue caused loss of receptor function. This identified 60 (15%) of the 410 missense SNVs in the GPCR gene target regions as potentially damaging (Suppl. Table 3, available online at www.thrombosis-online.com). Seven stop-gain SNVs were also classified as potentially damaging by manual annotation since they predicted protein truncation.

The total number of all classes of SNV that were classified as potentially damaging by either computational or manual annotation was 156 (21% of all SNVs; Suppl. Table 3, available online at www.thrombosis-online.com). Forty missense SNVs were classified as potentially damaging by both computational and manual annotation (Suppl. Table 2, available online at www.thrombosis-on line.com).

### Distribution of damaging missense GPCR gene variants

**Figure 2:**
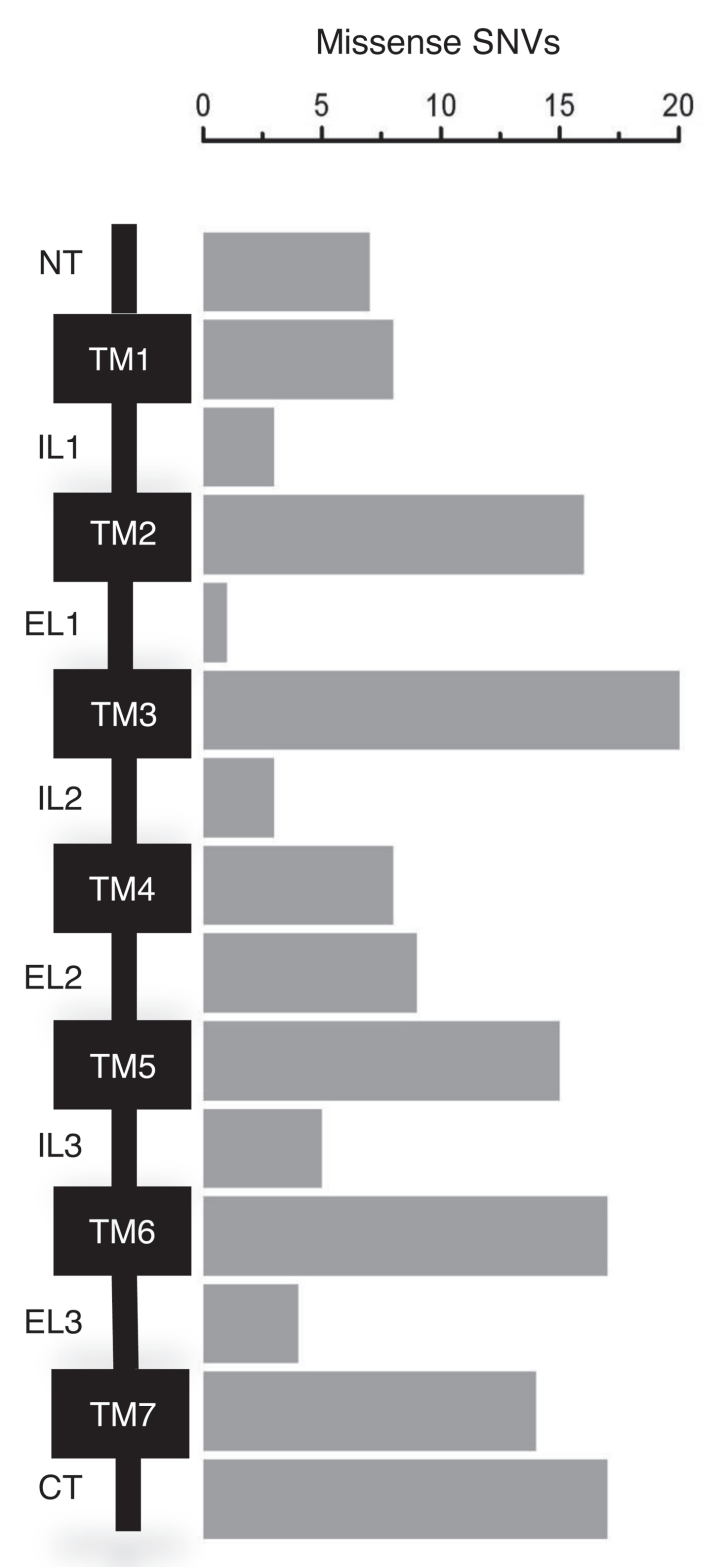
**Distribution of potentially damaging missense SNVs across GPCR domains.** Schematic of a prototypical Class A GPCR showing the N-terminal extracellular region (NT), the transmembrane helices (TM1–7), the intracellular (IL) and extracellular (EL) loops, and the C-terminal intracellular region (CT). The number of missense SNVs in the population databases classified as potentially damaging within these regions are represented in the bar chart.

The 149 potentially damaging missense SNVs were represented in all of the 18 selected GPCR genes (► Figure 1B) and predicted amino-acid substitutions that were more common in the TM domains and C-terminal intracellular region (CT) than other regions (► Figure 2). Twenty-five SNVs predicted amino-acid substitutions at sites shown in the consensus Class A GPCR structure to contribute to inter-helical interactions between the TM domains. A further 15 were in regions implicated in G protein interactions, five were in the helical regions of consensus ligand binding pockets, three were located in D/NPXXY motifs and one was in an E/DRY motif (► Table 2).

### Characteristics of patients with inherited platelet function disorders

The IPFD collection comprised 31 unrelated cases (11 males and 20 females; age range 6–82 years) with abnormal platelet function determined by light transmission aggregation and ATP release assays (19, 20). The collection comprised cases in which the main laboratory defect was within the Gi-pathway (n=15), secretion pathway (n=11) and thromboxane synthesis pathway (n=3) according to previous diagnostic criteria (19). Two cases showed complex defects that could not be classified. This collection was selected as a representative sub-group of a larger collection of 111 previously reported cases with inherited platelet function disorders enrolled into the UK GAPP study and showed a similar distribution of pathway defects to the group as a whole (19).

### GPCR gene variations in cases with inherited platelet disorders

Among the 31 cases with IPFD, we identified 256 SNVs in the target regions of the genes encoding the stimulatory platelet GPCRs PAR1, P2Y_12_, TPα, LPA_5_, CXCR4, PAR4, P2Y_1_, α_2A_-adrenoceptor, CCR4, V_1A_ receptor, PAF receptor, FPR1, EP3 receptor, succinate, and 5-HT_2A_ receptor. These comprised 38 individual SNVs of which 22 (58.9%) were synonymous and 16 (42.1%) were missense. There were no stop-gain or splice region SNVs. Thirty four unique SNVs were present in the 1,000 genomes and ESP population datasets (12 with global MAF<1% and 22 with global MAF ≥ 1%) and four were undocumented. Using an identical strategy to the analysis of the population datasets, we classified three heterozygous missense SNVs as potentially damaging in the IPFD cases, all within *P2RY12* (► Table 3). A wider analysis of variants identified in other platelet genes did not identify any single candidate variants that could completely account for the platelet phenotype of each case.

### Characteristics of cases with P2Y_12_ variants

**Figure 3:**
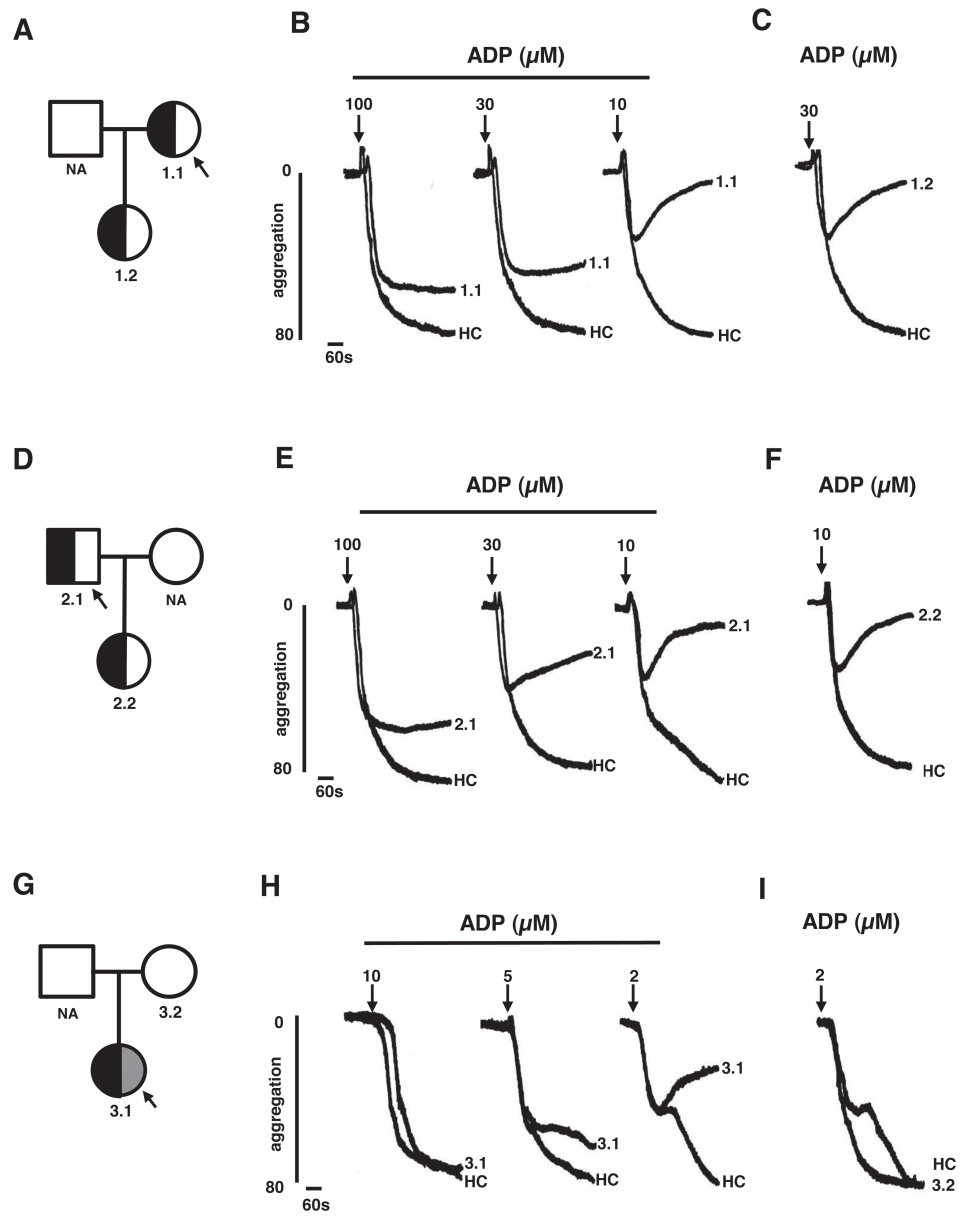
**Platelet phenotype in cases with potentially damaging SNV in P2RY12.** A) The pedigree of index case 1.1, who was heterozygous for the P2Y_12_ R122H substitution (half back shading), showing pedigree member 1.2, also with the P2Y_12_ R122H substitution. B) Light transmission aggregation (LTA) responses to 10–100 µM ADP in platelet-rich plasma from 1.1 and a healthy control (HC). C) LTA responses to 30 µM ADP in 1.2. D) The pedigree of index case 2.1, who was heterozygous for the P2Y_12_ P258T substitution (half black shading) showing pedigree member 2.2, also with the P2Y_12_ P258T substitution. E) LTA response to 10–100 µM ADP in platelet-rich plasma from 2.1 and an HC. F) LTA responses to 30 µM ADP in 2.2 and a HC. G) The pedigree of the index case 3.1 who was heterozygous for the P2Y_12_ V207A and T223R substitutions (indicated by half black and half grey shading), showing pedigree member 3.2 with wild-type P2Y_12_. H) LTA response to 2–10 µM ADP in platelet-rich plasma from 1.1 and an HC. I) LTA responses to 2 µM ADP in 3.2 and a HC.

The P2Y_12_ R122H variant was identified in a female index case 1.1 (►Figure 3A) with a history of prolonged bleeding from minor wounds and after a vaginal delivery. There was no abnormal bleeding after two other vaginal deliveries or after tonsillectomy. The P2Y_12_ P258T variant was identified in an unrelated male index case 2.1 (► Figure 3D) who had experienced recurrent gastrointestinal bleeding throughout adulthood but had no other bleeding symptoms. Platelets from case 1.1 and from case 2.1 showed normal shape change but reduced aggregation responses to 10–100 µM ADP compared to healthy controls that was reversible with 10 µM ADP (► Figure 3B and E), indicating selective loss of P2Y_12_ function. Compared to control subjects, platelets from both cases also showed reduced aggregation responses to 3–30 µM epinephrine and 1 µg/ml collagen, but not to 3 µg/ml collagen, which are consistent with loss of P2Y_12_ function. However, there were also reduced aggregation responses to 0.5–1 mM arachidonic acid in case 1.1 and reduced responses to ristocetin 1.25–1.5 mg/ml and a markedly reduced response to high concentration (100 µM) epinephrine in case 2.1. The latter findings indicate that cases 1.1 and 2.1 have distinct and complex aggregation phenotypes, neither of which can be completely explained by loss of P2Y_12_ function. Platelets from other pedigree members 1.2 and 2.2, analysed in parallel with the respective index cases, also showed reduced aggregation responses to ADP compared with controls (► Figure 3C and F). Cases 1.2 and 2.2 were subsequently shown to harbour the R122H and P258T variations respectively, but neither had abnormal bleeding symptoms.

The P2Y_12_ V207A variant was identified in an asymptomatic female index case 3.1 (► Figure 3G) who also harboured a P2Y_12_ SNV on the same allele that predicted a T223R substitution, that was classified as benign. Platelets from 3.1 showed normal platelet shape change but reduced aggregation responses to 5–20 µM ADP (► Figure 3H) and 5–10 µM epinephrine compared to controls. The maximum amplitude of responses to ADP and epinephrine were within the reference interval of responses determined from a panel of 30 locally recruited healthy controls, but fell within the lowest 10^th^ percentile of control responses, consistent with reduced P2Y_12_ function, that was less pronounced than index cases 1.1 and 2.1. Platelets from case 3.1 also showed reduced aggregation responses to 0.5–1 mM arachidonic acid suggesting an additional platelet defect. A pedigree member 3.2 with wild-type P2Y_12_ showed platelet aggregation responses to ADP that were similar to control subjects (► Figure 3I).

### Analysis of the P2Y_12_ R122H and V207A in HEK-293 cells

**Figure 4:**
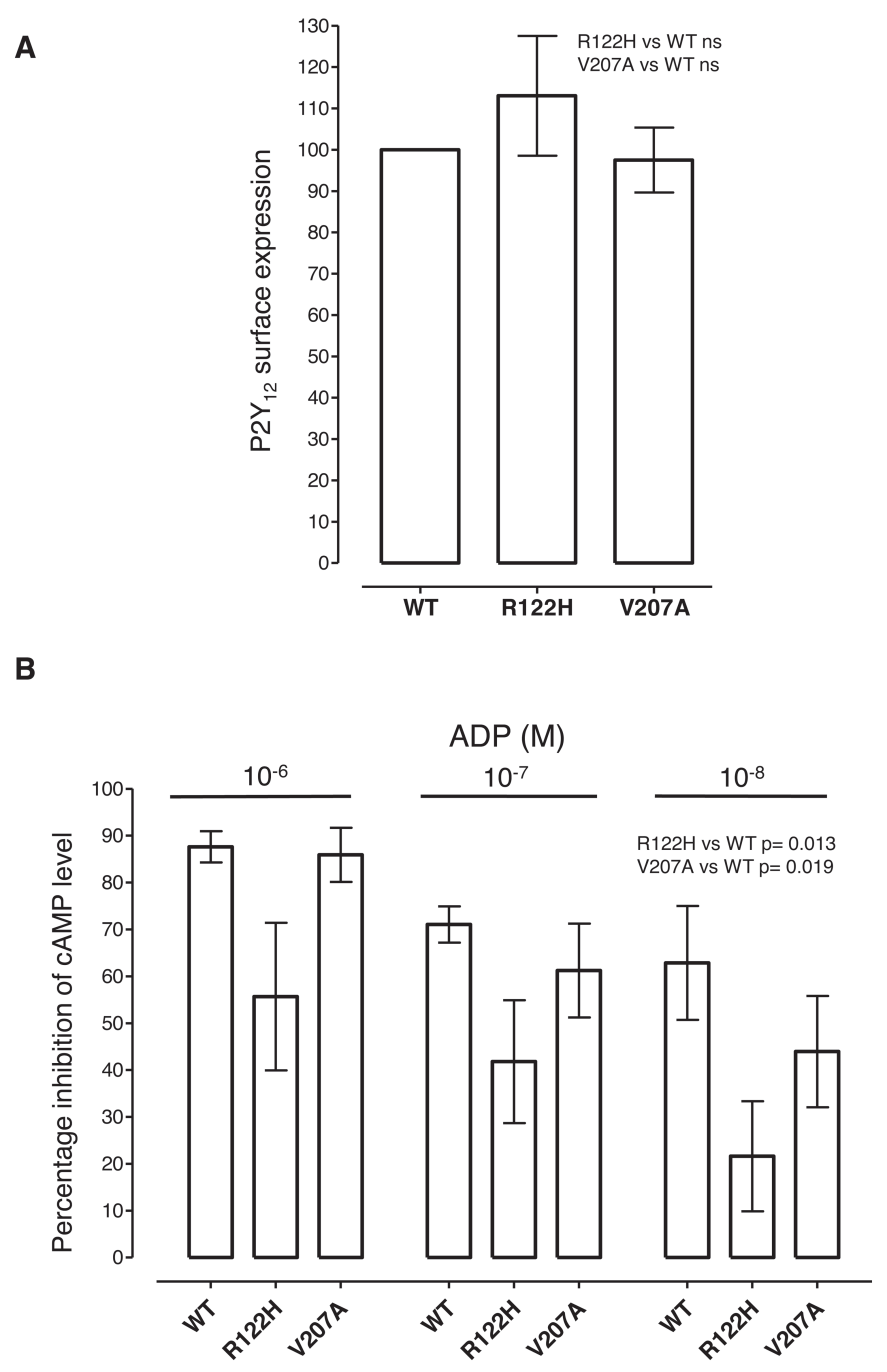
**Cell surface expression and function of P2Y_12_ R122H and V207A in transfected cells.** A) Cell surface expression of HA-tagged P2Y_12_ wild-type (WT), R122H and V207A was determined in transfected cells by ELISA using an anti-HA monoclonal antibody. Mean expression levels ± SEM in three independent experiments are presented, normalised to expression of WT P2Y_12_. B) The ability of P2Y_12_ WT, R122H and V207A to decrease cellular cAMP levels was determined in transfected cells by measuring residual cAMP by ELISA after incubation with the indicated concentrations of ADP. Data from three independent experiments are presented as the percentage inhibition (mean ± S. E. M) of the basal cAMP level in forskolintreated cells.

Since the R122H and V207A substitutions had not been previously associated with P2Y_12_ receptor deficiency in humans, we examined the phenotype of these substituted P2Y_12_ receptors in transfected cells. Expression of P2Y_12_ R122H and V207A was observed predominantly at the cell surface by immunofluorescence microscopy (data not shown). When cell-surface expression was quantified by ELISA, the normalised expression levels of the substituted receptors were almost identical to that of wild-type receptor (R122H mean 113% ± S. E. M. 14.5% and V207A 97.5% ± 7.9%; ► Figure 4A) indicating that neither substitution significantly affected P2Y_12_ receptor trafficking. When P2Y_12_ receptor function was tested by measuring the ability of ADP to reduce cellular cAMP levels, the substituted P2Y_12_ receptors showed less reduction in cAMP at ADP concentrations of 1 µM to 10nM compared to P2Y_12_ wild-type (p=0.013 for R122H and p=0.019 for V207A; 1 way ANOVA: ► Figure 4B). These data indicate that both substitutions reduce P2Y_12_ function, with a weaker effect from P2Y_12_ V207A substitution, consistent with the less marked platelet aggregation defect.

## Discussion

We have reported the results of a unique survey of coding and splice region SNVs in 18 platelet GPCR genes from 7,595 exomes in the 1,000 genomes and ESP databases and from 31 cases with IPFD. Our main findings were that: (i) in the population databases, the GPCR gene target regions contained potentially damaging SNVs that were individually rare, but collectively numerous; ii) the potentially damaging SNVs were diverse and were predicted to alter GPCR activity through several mechanisms, and, iii) a representative collection of cases with IPFD also had SNVs in platelet GPCR genes, including potentially damaging variants affecting P2Y_12_ in three cases.

Our strategy for identifying potentially damaging SNVs was based on computational annotation using six bioinformatic tools with different methodologies (22), complemented by manual annotation using resources that are unique to GPCRs. These included the GPCRDB database that catalogues previous GPCR mutagenesis experiments, the high resolution structures for the PAR1, A_2A_ and P2Y_12_ receptors (26–28) and the consensus structure for Class A GPCRs (25) that provides structural data for GPCRs with unsolved crystal structures. Combined computational and manual annotation has provided a valuable insight into the diversity and impact of rare variants in human GPCR genes. However, our analysis has focussed on missense rather than synonymous coding region SNVs. Since 6% of all synonymous SNVs in the ESP exome dataset were computed to be potentially damaging, primarily through codon usage effects (10, 29), our analysis is likely to have underestimated the overall burden of GPCR gene variation.

Within the ESP and 1,000 genomes databases, we found 740 SNVs in the GPCR gene target regions, of which 56% were missense and 70% had a global MAF<0.05% or were singleton records. These characteristics are similar to the entire ESP exome dataset comprising > 500,000 SNVs, of which 58% are missense and 72% are present in only three alleles or less (10), indicating an exome-wide abundance of rare missense variants. Our prediction that 21% of SNVs in the GPCR gene target regions were potentially damaging, is also similar to exome-wide estimates of 17% determined by computation (10). One noteworthy finding from our survey is that the platelet GPCR genes contained a median of 41.5 SNVs per coding region, compared with 24 SNVs per coding region exome-wide (10). The high variation rate in GPCR genes cannot be explained by differences in the length of coding region because the GPCR genes had median coding length 1121 bp, similar to the exome median of 1,100 bp (30). However, this difference could be related to GC content (31) which was 56.4% in platelet GPCR genes compared to 51% exome-wide (32). Consistent with this, the GPCR genes *F2RL3* and *PTGIR* with high GC content, had more SNVs than others with lower GC content, although this trend was inconsistent across all platelet GPCRs. We also showed that SNVs classified as potentially damaging were more common in gene regions encoding GPCR TM helices and CT intracellular regions compared to other areas. This reflects the essential roles of the TM helices in maintaining GPCR tertiary structure and defining the ligand binding and G-protein interaction sites (25) and the CT intracellular regions in regulating GPCR signalling and trafficking (33).

Consistent with the population databases, the 31 cases with IPFD also harboured rare missense SNVs in genes encoding the stimulatory platelet GPCRs. Although these were represented in all of the target GPCR genes, the three variants that were predicted to be potentially damaging were exclusively in *P2RY12* and occurred as heterozygous traits.

These included the P2Y12 P258T substitution which occurs adjacent to Y259 in TM6 that is required for ligand binding (28). This substitution was identified and characterised previously in an unrelated IPFD pedigree who also displayed reduced platelet responses to ADP (34), identical to the phenotype in the P2Y12 P258T pedigree in our study. Since this independent data provides good evidence that the P258T variation is causally related to loss of P2Y12 receptor function, we performed no further characterisation.

The other observed P2Y12 variants had not been previously reported. These included an SNV predicting an R122H substitution within the P2Y12 DRY motif which has multiple postulated roles in regulating receptor conformation, G-protein interactions and receptor trafficking (35). This substitution also occurs at a residue affected by a different substitution (P2Y12 R122C) in a previously reported IPFD pedigree with P2Y12 dysfunction (17). The P2Y12 V207A substitution affects a residue not previously associated with an IPFD, but which is adjacent to C208 in TM5 which has multiple interactions with TM3 and is, thereby, required for receptor structural integrity (25). Consistent with these significant structural predictions, we confirmed that the P2Y12 V207A and R122H substitutions were responsible for loss of P2Y12 receptor function by demonstrating diminished ADP-mediated reduction in cytoplasmic cAMP levels in transfected cells, which is a sensitive and highly specific measure of P2Y12 function (36). Our demonstration that cell surface expression of the substituted P2Y12 receptors was the same as wild-type suggest that both V207A and R122H disrupt function by impairing ligand binding, receptor activation or signal transduction, rather than by affecting receptor trafficking.

It is noteworthy that in the IPFD cases in this series, the R122H and P258T substitutions, and to a less pronounced extent the V207A substitution, were associated with reduced platelet responses to ADP consistent with impaired P2Y12 function. Platelet responses to low concentrations of other activating agonists were also reduced, in keeping with impaired P2Y12-mediated positive feedback from ADP released via dense granules, and similar to previous IPFD cases with loss-of-function P2Y12 variants (11, 34, 37). Despite this platelet phenotype, there was an inconsistent relationship between the heterozygous P2Y12 variants and abnormal bleeding, suggesting that partial loss of P2Y12 function alone is insufficient to affect haemostasis. It is also noteworthy that in all three index cases with potentially damaging P2Y12 variants, there were abnormal responses to other activating agonists that may not be explained solely by loss of P2Y12 function because of the magnitude of the other defects. Our data do not allow us to exclude a dominant negative effect from the observed heterozygous P2Y12 variants, or a further non-coding P2Y12 variant in trans that reduces expression of the other allele. However, a more plausible explanation is that in addition to a variant affecting P2Y12, the IPFD cases also harboured loss-of-function variants in other platelet genes that contributed to bleeding and to the complex laboratory phenotypes. The concept that some IPFD have complex heritability is supported by previous descriptions of pedigrees with phenotypes that are the composite effect of independent variants affecting P2Y12 and PAR1(17) and P2Y12 and von Willebrand factor (11). We speculate that the apparent over-representation of P2Y12 defects in our case series and in these previous reports, may reflect that clinical diagnostic LTA agonist panels have greater sensitivity for defective stimulatory GPCRs in non-redundant feedback pathways such as P2Y12, than other GPCRs, thereby introducing a selection effect.

Through a systematic analysis of platelet GPCR genes, we have highlighted the burden of rare SNVs in the general population and in selected patients with IPFDs. The variety and burden of potentially damaging SNVs in the healthy population recruited into the 1,000 genome and ESP databases highlight the incomplete penetrance of these variants. This suggests the possibility that mild IPFDs are more frequent in the general population, but may commonly go unnoticed until a challenge, such as childbirth, surgery or initiation of antiplatelet therapy, is applied. The spread of bleeding manifestations even in patients diagnosed with IPFDs and harbouring identical SNVs also highlights the challenges of applying genetic screening with platelet function testing approaches to large populations. Rare variants affecting GPCR function may be solely responsible for the phenotypes of some isolated pedigrees with IPFD, but could also contribute to complex defects caused by variants in other platelet genes.

**What is known about this topic?**

- Common genetic variants associated with platelet G protein-coupled receptor (GPCR) genes influence receptor activity, but with small effect size.
- Rare GPCR gene variants have to date been reported in the P2Y_12_ and TP receptor genes in small numbers of pedigrees with inherited platelet function disorders.

**What does this paper add?**

- In population databases, GPCR genes contain numerous rare SNVs that are potentially damaging and occur at low individual allele frequencies.
- Rare SNVs in GPCR genes are also present in patients with inherited platelet function disorders and may contribute to the platelet laboratory phenotype.

## Supplementary Material

Suppl. Table 1, 2

Suppl. Table 3

## Figures and Tables

**Table 1:** **Platelet G-protein coupled receptors.** The table lists the 18 G protein-coupled receptors described in IUPHAR terminology, listed in order of decreasing transcript abundance in human megakaryocytes (18). †Principal coupled G protein. ‡Major CCDS annotated transcript with experimental evidence in megakaryocytes.

Gene Name	Receptor Name	GC content (%)	G-protein†	Protein Accession	Transcript Accession‡
*F2R*	PAR1	49.8	Gq	ENSP00000321326	ENST00000319211
*P2RY12*	P2Y_12_	40.7	Gi	ENSP00000307259	ENST00000302632
*PTGIR*	IP_1_	69.0	Gs	ENSP00000291294	ENST00000291294
*TBXA2R*	TPα	69.6	Gq	ENSP00000364336	ENST00000375190
*LPAR5*	LPA_5_	67.9	Gq	ENSP00000327875	ENST00000329858
*ADORA2A*	A_2A_ receptor	61.3	Gs	ENSP00000336630	ENST00000337539
*CXCR4*	CXCR4	50.3	Gi	ENSP00000241393	ENST00000241393
*F2RL3*	PAR4	69.9	Gq	ENSP00000248076	ENST00000248076
*P2RY1*	P2Y_1_	51.0	Gq	ENSP00000304767	ENST00000305097
*ADRA2A*	α_2A_-adrenoceptor	61.3	Gz	ENSP00000280155	ENST00000280155
*CCR4*	CCR4	48.2	Gi	ENSP00000332659	ENST00000330953
*PTGDR*	DP_1_ receptor	59.9	Gs	ENSP00000303424	ENST00000306051
*AVPR1A*	V_1A_ receptor	58.9	Gq	ENSP00000299178	ENST00000299178
*PTAFR*	PAF receptor	53.8	Gq	ENSP00000362965	ENST00000373857
*FPR1*	FPR1	52.7	Gi	ENSP00000471493	ENST00000304748
*PTGER3*	EP_3_ receptor	60.8	Gq	ENSP00000302313	ENST00000306666
*SUCNR1*	succinate receptor	43.0	Gi	ENSP00000355156	ENST00000362032
*HTR2A*	5-HT_2A_ receptor	46.0	Gq	ENSP00000437737	ENST00000378688

**Table 2:** **Potentially damaging missense variants in platelet GPCR genes in the 1,000 Genomes and ESP6500 databases.** The table lists the subgroup missense SNV classified as potentially deleterious by both manual and computational annotation. For each missense SNV the results of six independent annotation tools: P-SNP (the consensus classifier), MAPP, PhD-SNP, PP-1, PP-2, SIFT and SNAP are indicated as + for ‘damaging’ and – for ‘not damaging’. / indicates the tool did not return a prediction for the SNV.

GPCR	Variation	Substitution	Region	Manual annotation	P-SNP	MAPP	PhD-SNP	PP-1	PP-2	SIFT	SNAP
PAR1	rs144447562	Y266C	EL2	Tethered ligand binding region	+	/	+	+	+	-	+
PAR1	rs372280945	L354F	TM7	Consensus ligand binding pocket	+	/	+	-	+	+	-
P2Y_12_	rs372954515	T283I	TM7	TM6-TM7 conserved interaction	+	+	+	+	+	+	+
P2Y_12_	rs367926037	M108L	TM3	TM3-TM6 conserved interaction; ligand binding pocket	+	+	+	-	+	+	+
P2Y_12_	rs202099742	P258T	EL3	Identified in subject with IPFD	+	-	-	+	+	+	+
IP_1_	rs147448416	R77C	TM2	Reduced surface expression (reduced Bmax)	+	/	+	+	+	+	+
IP_1_	rs4987262	R212C	IL3	Reduced surface expression (reduced Bmax)	+	/	+	+	+	+	+
IP_1_	rs201340109	R215C	IL3	Reduced surface expression (reduced Bmax)	+	/	+	+	+	+	+
IP_1_	rs370909150	H237R	TM6	Site of G protein interaction within helices	+	/	+	+	+	+	+
TPα	rs34377097	R60L	ICL1	TM2-TM3 conserved interaction	+	+	+	-	+	+	+
TPα	rs201421330	M126T	TM3	TM3-TM2 conserved interaction	+	+	+	-	+	+	+
TPα	rs372994525	S218G	TM5	Site of G protein interaction within helices	+	+	-	-	+	+	+
TPα	rs61731124	T286M	TM7	Consensus ligand binding pocket	+	-	-	+	+	+	+
TPα	rs374635591	P305L	TM7	TM1-TM7 conserved interaction	+	+	+	+	+	+	+
TPα	rs370418735	R326W	CT	Serine phosphorylation site	+	-	-	+	+	+	-
LPAR_5_	rs149664830	M103K	TM3	TM3-TM4 conserved interaction	+	+	-	+	-	+	+
LPAR_5_	rs187536858	P294L	TM7	TM1-TM7 conserved interaction	+	+	+	+	+	+	+
LPAR_5_	rs374800227	Y298H	TM7	TM7-TM2 conserved interaction	+	+	-	+	+	+	+
A_2A_	rs142560733	R293C	CT	Minus 5 from Thr phoshorylation site	+	+	+	+	+	+	+
CXCR4	rs368016542	D84H	TM2	TM1-TM2 conserved interaction	+	+	-	+	+	+	+
CXCR4	rs367718547	D193Y	EL2	Mediates dimerization	+	-	+	+	+	+	-
PAR4	rs374965245	G13E	NT	Signal peptide	+	-	-	+	+	+	+
PAR4	rs201697829	R47H	NT	Site of cleavage of tethered ligand	+	/	-	+	+	+	-
PAR4	rs2227346	F296V	TM6	TM3-TM6 conserved interaction; ligand binding pocket	+	+	-	+	+	+	+
PAR4	rs111890288	S329C	TM7	Consensus ligand binding pocket	+	-	+	-	+	+	-
α_2A_	rs149350078	I234M	TM5	Site of G protein interaction within helices	+	+	+	+	+	+	+
α_2A_	rs370313798	R383G	TM6	Site of G protein interaction within helices	+	+	+	+	+	+	+
α_2A_	rs375454021	V390M	TM6	Site of G protein interaction within helices	+	+	-	+	+	+	-
CCR4	rs200003145	A240V	TM6	Site of G protein interaction within helices	+	-	+	+	+	+	-
DP_1_	rs145604058	T69M	TM2	TM1-TM2 conserved interaction	+	+	-	-	+	+	-
DP_1_	rs370262391	A127T	TM3	TM3-TM5 conserved interaction	+	+	+	-	+	+	+
V_1A_	rs180760072	F133C	TM3	TM3-TM4 conserved interaction	+	-	+	-	+	+	-
V_1A_	rs369710823	M145T	TM3	TM3-TM2 conserved interaction	+	-	+	-	-	-	-
V_1A_	rs377107751	V153L	TM3	Site of G protein interaction within helices	+	+	+	-	-	+	-
PAF	rs139524224	R229Q	TM6	Site of G protein interaction within helices	+						
FPR1	rs369354920	D122H	TM3	E/DRY motif	+	+	-	+	+	+	+
FPR1	rs149931707	R123H	TM3	Site of G protein interaction within helices	+	+	-	+	+	+	+
FPR1	rs368075541	R238H	TM6	Site of G protein interaction within helices	+	-	-	-	+	+	-
succinate	rs371440145	N41K	TM1	TM1-TM2 conserved interaction	+	+	+	+	+	+	+
succinate	rs374436394	I240N	TM6	TM3-TM6 conserved interaction	+	+	-	+	+	+	+
succinate	rs142256005	R252Q	TM6	Agonist dependent activation	+	-	+	+	+	+	-
succinate	rs142852744	R291Q	TM7	Agonist dependent activation; TM6-TM7 interaction	+	-	+	+	+	+	+
5-HT_2A_	rs375024989	N38K	NT	N-linked glycosylation site	+	-	-	+	+	+	+

**Table 3:** **Potentially damaging missense SNV in P2RY12 in cases with IPFD.** This table lists the missense SNV found in P2RY12 in cases with a demonstrable platelet aggregation defect. For each missense SNV the results of six independent annotation tools: P-SNP (the consensus classifier), MAPP, PhD-SNP, PP-1, PP-2, SIFT and SNAP are indicated as + for ‘damaging’ and – for ‘not damaging’.

Substitution	Variation	Manual annotation	P-SNP	MAPP	PhD-SNP	PP-1	PP-2	SIFT	SNAP
R122H	novel	DRY motif	+	+	+	+	+	+	+
P258T	rs202099742	TM6; adjacent to Y259 that contributes to ligand binding	+	-	-	+	+	+	+
V207A	rs370983746	TM5; adjacent to C208 that has multiple binding contacts with TM3	+	+	-	-	+	+	+
