# Supplementary material for: Diversity and Impact of Rare Variants in Genes Encoding the Platelet G Protein-Coupled Receptors
Source: Thromb Haemost. 2015 Jan 8;113(4):826–37. doi: 10.1160/TH14-08-0679 (PMC4510585; doi:10.1160/TH14-08-0679)
Supplement: Suppl. Table 1, 2 [file TH-113-04-14080679-s001.pdf]

Supplementary Material to Jones et al. "Diversity and impact of rare variants in genes encoding the platelet G protein-coupled receptors" (Thromb Haemost 2015; 113.3)

Suppl. Table 1: URLs utilised in GPCR gene selection and analysis.

Ensembl Genome Browser	http://www.ensembl.org/
Platelet Web	http://plateletweb.bioapps.biozentrum.uni-wuerzburg.de/plateletweb.php
BioMart Reource	http://www.ensembl.org/biomart/martview/
UniProt	http://www.uniprot.org/
GPCRDB	http://www.gpcr.org/7tm/
PredictSNP	http://loschmidt.chemi.muni.cz/predictsnp/
IUPHAR GPCR Database	http://www.guidetopharmacology.org/GRAC
Human Splice Finder	http://www.umd.be/HSF/

Suppl. Table 2: Functionally significant motifs or key structural residues in target GPCRs.

Feature	Description	Position of key interactions
**The inter-TM helix scaffold**	a conformation-independent , non-covalent network of interactions conserved amongst GPCRs	36 positions are involved; $Asn^{1.50}$, $Asp^{2.50}$, $Trp^{4.50}$, $Pro^{7.50}$ are highly conserved across class A GPCRs
**TM3 structural hub**	TM3 is an essential hub for the inter-helical network of conserved interactions	Almost all positions in TM3 participate in the consensus binding network; a conserved disulphide bond $Cys^{3.25}$ & Cys in ECL2
**Ligand binding cradle**	the extracellular face of TM helices that form the bottom of the ligand binding pocket	positions 3.32, 3.33, 3.36, 6.48, 6.51 & 7.39
**Intracellular loop 2 (IL2)**	contains the E/DRY motif that participates in the 'ionic lock' in some GPCRs	Arg in ICL2 forms salt bridge with $Asp^{3.49}$ of the E/DRY motif in TM3 in several receptors; $Arg^{3.50}$ of the E/DRY motif interacts with $Glu^{6.30}$ to form the ionic lock in some GPCRs; interacts with G protein after activation.
**G-protein binding region**	the intracellular face of the TM helices that make contact with G-proteins	positions 3.50, 3.53, 3.54, 5.61, 5.64, 5.65, 6.33 & 6.36 (based upon overlap between rhodopsin-G_t peptide and β_2-AR-G_s structures)
**The transmission switch**	rearrangement of these residues upon activation	positions 3.40, 5.51, 6.44 & 6.48

Suppl. Table 3: Master list of all damaging SNVs in population data set.

See separate file TH113.3_Jones_Suppl_Table3
